# Intra-Tumoral Nerve-Tracing in a Novel Syngeneic Model of High-Grade Serous Ovarian Carcinoma

**DOI:** 10.3390/cells10123491

**Published:** 2021-12-10

**Authors:** Jeffrey L. Barr, Allison Kruse, Anthony C. Restaino, Natalia Tulina, Sarah Stuckelberger, Samuel J. Vermeer, Caitlin S. Williamson, Daniel W. Vermeer, Marianna Madeo, Jillian Stamp, Maria Bell, Mark Morgan, Ju-Yoon Yoon, Marilyn A. Mitchell, Anna Budina, Dalia K. Omran, Lauren E. Schwartz, Ronny Drapkin, Paola D. Vermeer

**Affiliations:** 1 Cancer Biology and Immunotherapies Group, Sanford Research, 2301 East 60th St. North, Sioux Falls, SD 57104, USA; Jeffrey.Barr@sanfordhealth.org (J.L.B.); allisonkruse_2010@hotmail.com (A.K.); Anthony.Restaino@sanfordhealth.org (A.C.R.); Caitlin.Williamson@sanfordhealth.org (C.S.W.); dvermeer@sabbiotherapeutics.com (D.W.V.); Marianna.madeo@gmail.com (M.M.); Jillian.Stamp@sanfordhealth.org (J.S.); 2 Sanford School of Medicine, University of South Dakota, 414 East Clark St., Vermillion, SD 57069, USA; 3 Penn Ovarian Cancer Research Center, Department of Obstetrics and Gynecology, Division of Gynecologic Oncology, Perelman School of Medicine, University of Pennsylvania, 421 Curie Blvd, Philadelphia, PA 19104, USA; ntulina@pennmedicine.upenn.edu (N.T.); sarah.stuck@bluewin.ch (S.S.); mark.morgan@pennmedicine.upenn.edu (M.M.); mam@pennmedicine.upenn.edu (M.A.M.); domran@pennmedicine.upenn.edu (D.K.O.); rdrapkin@pennmedicine.upenn.edu (R.D.); 4 Lincoln High School, 2900 South Cliff Avenue, Sioux Falls, SD 57105, USA; SJVermeer26@gmail.com; 5 Sanford Gynecologic Oncology, Sanford Health, 1309 West 17th St., Sioux Falls, SD 57104, USA; Maria.Bell@sanfordhealth.org; 6 Laboratory Medicine, Department of Pathology, Perelman School of Medicine, University of Pennsylvania, 3400 Spruce St., Philadelphia, PA 19104, USA; Ju-Yoon.Yoon@unityhealth.to (J.-Y.Y.); budina@pennmedicine.upenn.edu (A.B.); lauren.schwartz@pennmedicine.upenn.edu (L.E.S.)

**Keywords:** ovarian cancer, innervation, ultrasound, nerve-tracing

## Abstract

Dense tumor innervation is associated with enhanced cancer progression and poor prognosis. We observed innervation in breast, prostate, pancreatic, lung, liver, ovarian, and colon cancers. Defining innervation in high-grade serous ovarian carcinoma (HGSOC) was a focus since sensory innervation was observed whereas the normal tissue contains predominantly sympathetic input. The origin, specific nerve type, and the mechanisms promoting innervation and driving nerve-cancer cell communications in ovarian cancer remain largely unknown. The technique of neuro-tracing enhances the study of tumor innervation by offering a means for identification and mapping of nerve sources that may directly and indirectly affect the tumor microenvironment. Here, we establish a murine model of HGSOC and utilize image-guided microinjections of retrograde neuro-tracer to label tumor-infiltrating peripheral neurons, mapping their source and circuitry. We show that regional sensory neurons innervate HGSOC tumors. Interestingly, the axons within the tumor trace back to local dorsal root ganglia as well as jugular–nodose ganglia. Further manipulations of these tumor projecting neurons may define the neuronal contributions in tumor growth, invasion, metastasis, and responses to therapeutics.

## 1. Introduction

Ovarian cancer, the fifth most common cancer in women, remains the most lethal gynecologic malignancy [1]. While this heterogeneous disease consists of multiple histological subtypes, high-grade serous ovarian carcinoma (HGSOC) accounts for 70% of the cases and the majority of the deaths [1]. Worldwide, nearly 300,000 women are diagnosed with this disease annually, half of which will succumb within the first 12 months [2]. This poor prognosis is commonly attributed to non-specific symptoms and a lack of screening and early detection [3,4]. Together, these factors typically culminate in late-stage (metastatic) diagnosis. 

Preclinical mouse models are essential tools for investigating the processes of tumor development, growth, and disease progression. For ovarian cancers, preclinical modeling is particularly urgent given its poor prognosis. Moreover, standard-of-care treatment consists predominantly of chemotherapy. While some ovarian cancer patients benefit from new therapeutics (targeted, immunotherapies), the majority do not [5]. The emergence of faithful pre-clinical models provides a means with which to test new therapeutics or therapy combinations to advance treatment for this aggressive disease. Given the urgent need for such models, it is not surprising that syngeneic models harboring known human alterations, replicating the ovarian cancer landscape, tumor microenvironment and development of ascites have recently been published [6,7,8]. Here, we describe a syngeneic model of HGSOC derived from oviducts, the cell of origin of this disease [9,10,11,12]. In addition to utilizing oviductal cells, this model was also engineered to include loss of *Trp53* and *Pten*, commonly altered in cases of HGSOC. Together, these features generate a murine model that is faithful to the human disease. Thus, we utilize this system to characterize tumor innervation. 

The presence of intra-tumoral nerves and the growing understanding of their active contributions to cancer initiation and progression illustrates the complexity of the tumor microenvironment (TME) and offers the possibility of new targets for therapeutic intervention [13]. Recent studies indicate a significant role for autonomic (sympathetic and parasympathetic) and sensory innervation of the TME in the regulation of tumor cell growth, migration, and invasiveness [14,15,16,17]. Interestingly, a decrease in tumor growth occurs following sympathetic and sensory denervation in models of pancreatic and breast cancer as well as melanoma [18,19,20], while parasympathetic denervation generates mixed effects [21,22]. The contributions of different autonomic and sensory nerve populations vary by cancer type and may depend on the anatomical location where tumorigenesis occurs. The ovary receives innervation from sympathetic sources as wells as dorsal root ganglion (DRG) [23,24,25]. Moreover, innervation of the female reproductive tract undergoes marked plasticity associated with hormonal changes and reproductive status [26]. While the presence of nerves in these tissues is understood, studies documenting innervation of ovarian tumors and the source of these intra-tumoral nerves is lacking.

Neural tracing has been a valuable tool for defining circuits within the central nervous system (CNS). We have implemented axonal tracing with the fluorescent marker wheat germ agglutinin (WGA) to study the presence and source of intra-tumoral nerves in HGSOC. WGA is a lectin with specific affinity for neural membranes, binding to specific saccharide components of glycoproteins and glycolipids ubiquitous in neuronal membranes. Moreover, neurons have lectin receptors concentrated at axon terminals [27,28,29]. Therefore, once taken up, this neuro-tracer will label only neurons with terminals at the site of injection. In addition, fluorescent retrograde tracers do not require additional immunohistochemical processing and can be transported transynaptically or transganglionically [30,31,32] enabling extensive circuit mapping. Additional advantages to using WGA is its rapid transport in vivo, its intense brightness, which facilitates whole mount imaging of tissue, and its flexibility for pairing with other markers [33]. Here, we identify that HGSOCs are innervated, describe a new syngeneic model of HGSOC, and provide evidence utilizing this model that the source of tumor innervation is via recruitment from regional nerve endings originating from peripheral ganglia. 

## 2. Materials and Methods

### 2.1. Immunohistochemistry (IHC) 

Tissues were obtained with Institutional Review Board approval from Sanford Health and the University of Pennsylvania through the BioTrust Collection (https://www.med.upenn.edu/OCRCBioTrust/ accessed on 1 September 2021). Tissues were fixed in 10% neutral buffered formalin and processed on a Leica 300 ASP tissue processor. Tissue sections (5 µm) were immunohistochemically stained for β-III tubulin (2G10, ab78078, 1:250, Abcam; RRID: AB_2256751), TRPV1 (cat# ACC-030, 1:100, Alomone labs; RRID: AB_2313819), TH (Ab112, 1:750, Abcam; RRID: AB_297840), and VIP (ab22736, 1:100, Abcam; RRID: AB_447294); sections were also histochemically stained by hematoxylin & eosin. Antibody optimization and staining were performed with the BenchMark^®^ XT automated slide staining system (Ventana Medical Systems, Inc., Oro Valley, AZ, USA). Primary antibody was omitted as the negative control. For hematoxylin & eosin staining, slides were stained on a Sakura Tissue-Tek H & E stainer. The program runs as follows: deparaffinize and rehydrate tissue, stain in Gill’s hematoxylin (2 min), differentiate running tap water, blue in ammonia water, and counterstain in eosin (1 min), dehydrate and clear. Slides were counterstained with hematoxylin, dehydrated, cleared, and coverslipped. The Aperio VERSA 8 slide scanning system from Leica Biosystems, equipped with a Point Grey Grasshopper3 color camera for brightfield scanning was used to analyze stained sections. 

### 2.2. Scoring of IHC Staining 

Five independent evaluators scored all tissue samples at 20× magnification on an Olympus BX51 microscope, scoring 5 random fields/sample for β-III tubulin labeled nerve twigs. A score of 0 was given to indicate the absence of staining within each field; a score of +1 indicated 1–10% staining, +2 indicated 30–50% staining, and +3 indicated greater than 50% staining. Only single twigs were scored; nerve bundles were not scored.

### 2.3. Double Immunofluorescent Staining

Formalin fixed and paraffin-embedded sections were deparaffinized and rehydrated by using the following washes at RT: 100% Histo-Clear (National Diagnostics) for 5 min, 100% ethanol for 1 min, 90% ethanol for 1 min, 70% ethanol for 1 min and then in PBS for 1 min. A heat-induced antigen retrieval step was performed prior to immunofluorescent staining as follows: sections were incubated with 10 mM Sodium Citrate Buffer (10 mM Sodium Citrate Buffer, 0.05% Tween 20, pH 6.0) at 95 °C for 1 h. After cooling down at room temperature for 30 min, slides were washed with PBS and then blocked in blocking buffer (1X PBS, 10% goat serum, 0.5% TX-100, 1% BSA) for 1 h at RT. Sections were incubated with primary antibodies [β-III tubulin (Abcam, cat# 78078, 1:100 dilution, RRID:AB_2256751), TRPV1 (Alomone labs, cat# ACC-030, 1:100 dilution, RRID:AB_2313819), neurofilament (Biolegend, cat#837801, 1:100, RRID:AB_2565383)] overnight at +4 °C. Slides were washed three times in PBS for 5 min each and incubated in secondary antibodies and Hoescht (1:10,000, Invitrogen) at RT. Slides were washed in PBS three times, for 5 min each, and coverslips were mounted by using Faramount^TM^ aqueous mounting media (Dako). Immunostained sections were observed by using an Olympus FV1000 confocal microscope equipped with a laser scanning fluorescence and a 12-bit camera; images were taken using a 60× or 100× oil PlanApo objective.

### 2.4. Ovarian Cancer Tumor Model

The *Trp53* ^-/-^ *Pten* ^-/-^ murine model of HGSOC was generated as follows. Ten oviducts were isolated from 6-week-old female mice (C57Bl/6) under aseptic conditions and treated with trypsin for 30 min at 37 °C. The resulting cell suspension was centrifuged at 400 rpm for 5 min at 4 °C and pelleted cells were re-suspended in α-MEM medium containing ribonucleosides, deoxynucleosides, and L-glutamine (Gibco; Cat#12571-048) and supplemented with 10 ug/mL insulin-transferrin-sodium selenite (Roche; #11074547001), 20 pg/mL β-estradiol (Sigma; # E8875), 10 u/mL penicillin-streptomycin solution (Invitrogen; #15140122) and 10% fetal bovine serum (Atlanta Biologicals; Cat#S11550). Most cultured mouse oviduct secretory epithelium derived cells (MOSEC) lacked cytoplasmic protrusions characteristic for ciliated cells and expressed common markers of secretory cells, Pax8 and OVGP1. Some ciliated cells were observed initially but were eliminated after successive cell passages, consistent with a similar protocol to isolate human fallopian tube secretory cells [34,35].

Exon 5 of the *Trp53* gene and the phosphatase domain of *Pten* were targeted using the CRISPR-Cas9 system in the second passage of cultured primary MOSEC. The synthetic guide (sg) RNAs, GAAGTCACAGCACATGACGGAGG and TGGTCAAGATCTTCACAGAA against *Trp53* and *Pten*, respectively, were generated by annealing respective crRNA and tracrRNA pairs according to manufacturer’s instructions (Invitrogen [36]. The cells were then transfected with the TrueCut Cas9 protein v2 (Invitrogen; Cat#A36496) and sgRNA complexes using the Lipofectamine CRISPRMAX Cas9 Transfection Reagent (Invitrogen; Cat#CMAX00008). The presence of mutations and loss of protein expression was confirmed by Sanger sequencing and Western blot analysis, respectively, in two different *Trp53* ^-/-^; *Pten* ^-/-^ double knockout lines (clones 2 and 4).

For initial characterization of the tumor model, *Trp53* ^-/-^; *Pten* ^-/-^ DKO MOSEC cells (clone 4) were expanded in culture and injected intraperitoneally (i.p.) into five 6-week-old C57Bl/6 female mice (1 × 10^7^ cells in ice cold PBS per animal). All animal testing was conducted in strict accordance with the recommendations of the National Institutes of Health set out in the Guide for the Care and Use of Laboratory Animals and with an approved protocol from University of Pennsylvania Institutional Animal Care and Use Committee. Tumor morphology was assessed using hematoxylin and eosin staining and immunohistochemical analyses with HGSOC markers (Pax8, WT-1) and was found to be consistent with that of HGSOC. Pax8 antibody (ProteinTech, Rosemont, IL, USA, 10336-1-AP, 1:3000) and WT-1 (Abcam, ab89901, 1:300) required a citrate buffer pressure cooker method of antigen retrieval. 

Tumor tissue isolated from tumor-bearing mice was dissociated using 90 µg/mL collagenase (GIBCO, Cat#17105-041), 500 µg/mL dispase (GIBCO, Cat#17105-041) and 1 µg/mL DNAse I (Sigma, Cat#D4527) in culture medium (α-MEM medium supplemented with ribonucleosides, deoxynucleosides, and L-glutamine (Gibco; Cat#12571-048) and containing 10 μg/mL insulin-transferrin-sodium selenite (Roche; #11074547001), 20 pg/mL β-estradiol (Sigma; # E8875), 10 u/mL penicillin-streptomycin solution (Invitrogen; #15140122) containing 10% fetal bovine serum (Atlanta Biologicals; Cat#S11550). Tumor-derived lines were developed and injected i.p. into 10 female C57Bl/6 mice. All animals developed tumors within five weeks of injection. Histological and immunohistochemical analyses of these tumors showed that they maintained HGSOC-like morphology and marker expression.

### 2.5. Preparation and Injection of Trp53 ^-/-^ Pten ^-/-^ Cell Lines for Innervation Studies

The *Trp53* ^-/-^ *Pten* ^-/-^ cells were grown in Minimum Essential Medium (MEM) alpha (500 mL, Gibco, 12571-048) with L-glutamine, supplemented with 10% Fetal Bovine Serum (FBS), 500 mL insulin/transferrin/sodium selenite (Roche Cat# 11074547001), 10 mL of beta-estradiol (Sigma Cat# E8875) maintained at 37 °C and 5% CO_2_ culture medium was refreshed every 3 days. Once the cells reached 85–90% confluence, the media was removed and cells were washed twice with warm (37 °C), sterile phosphate-buffered saline (PBS). Cells were trypsinized and suspended in cold Matrigel sufficient to yield 5 × 10^6^ cells/100 μL/mouse. Cells were kept cold until ready to inject and mixed before and in between injections to prevent cells from settling to the bottom of the tube, ensuring an accurate implantation of cells. Seven-week-old female C57Bl/6 mice (n = 4, Jackson laboratories, Bar Harbor, ME) were injected IP with 100 μL 5 × 10^6^ *Trp53* ^-/-^ *Pten* ^-/-^ cells. As a control, mice were similarly injected subcutaneously in the hind limb (n = 4). 

### 2.6. Ultrasound-Guided Injection of Axonal Tracer and Collection of Peripheral Ganglia

Starting at 1-week post-tumor implantation, the abdomen was scanned weekly to assess tumor formation by ultrasound (MS700 probe 48 Hz, Vevo2100, VisualSonics with abdominal imaging package) under isoflurane anesthesia. After locating the tumor region, the position of the transducer and surrounding anatomical structures of interest were noted. When tumors reached approximately 3 × 3 mm in size, they were injected with neuronal tracer as described below. 

Mice were anesthetized and prepped for imaging. A 10 µL Hamilton syringe with 30-G needle was loaded with WGA-Alexa 568 (1% in PBS) and placed bevel side up in the syringe clamp. The needle was slowly inserted through the skin and the peritoneal wall. The needle guide was used to visualize the needle tract to the target. The needle was advanced just past the middle of the tumor then pulled back slightly to reduce pressure and prevent leakage outside of the tumor. Tracer was slowly injected (2 μL over 10 min). The needle was maintained in place for 2 min after the infusion was complete and then slowly retracted from the mouse abdomen. Mice were quickly returned to a mouse cage on a heating pad until they fully recovered from anesthesia. Five days following intra-tumoral injection of neuronal tracer, mice were deeply anesthetized and transcardially perfused (PBS/4% paraformaldehyde). Using a stereo microscope, laminectomy was performed to remove the roof of the vertebral canal and expose the spinal cord and DRGs [37]. DRGs from all spinal levels were removed and collected in HBSS in a 96 well plate on ice. Tumor tissue and jugular–nodose ganglia, which contain the sensory peripheral neurons of the vagus nerve, where also collected [38]. Immunofluorescent staining was carried out for TRPV1, TH, and VIP as described above (Section 2.2). 

### 2.7. Human Studies

The cases for this study were obtained with patient consent and the study was approved by the Institutional Review Boards at Sanford Research and the University of Pennsylvania (through the BioTrust Collection; https://www.med.upenn.edu/OCRCBioTrust/accessed on 1 September 2021). Ovarian cancer cases utilized consisted of high-grade serous ovarian carcinoma (n = 75 formalin-fixed paraffin-embedded (FFPE) tumors). Control FFPE tissues were also collected (normal ovary: n= 10; normal fallopian tube: n = 10). Consented patients spanned 38–83 years of age. FFPE samples were cut into 5µm sections and immunohistochemically stained. Cases of breast, prostate, pancreatic, lung, liver, and colon cancers consisted of n = 10 for each cancer type. The breast cancer cases were all female and ranged in ages 43–86. The prostate cancer patient samples were all males ages 48–71. Pancreatic patient samples consisted of n= 6 females ages 48–90 and n = 4 males ages 73–79. Lung cancer patient samples consisted of n = 5 females ages 52–77 and n = 5 males ages 54–70. Liver cancer patient samples consisted of n= 6 females ages 45–84 and n = 4 males ages 56–74. Colon cancer patient samples consisted of n = 5 females ages 55–85 and n = 5 males ages 59–91. 

## 3. Results

### 3.1. Sensory Nerves Innervate HGSOCs 

**Figure 1:**
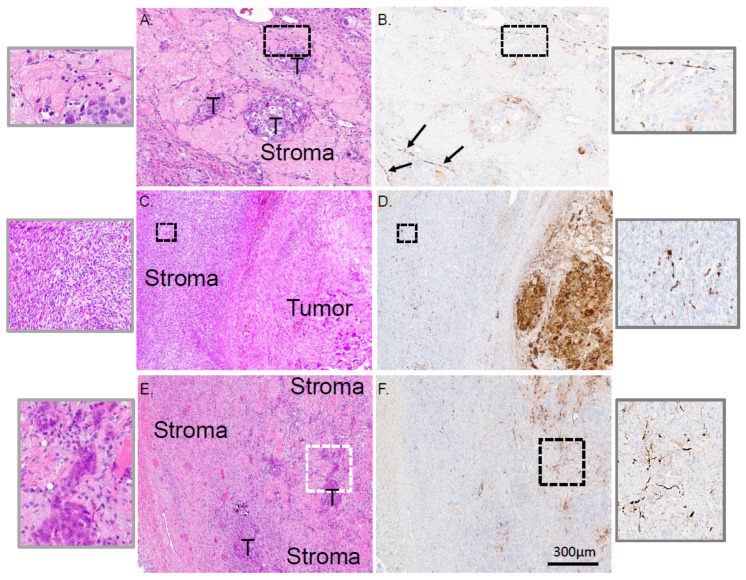
Representative serial sections of three HGSOC cases with serial sections histologically stained by H&E and immunohistochemically stained for β-III tubulin. In the first example (**A**,**B**) tumor islands (T) are surrounded by stroma. β-III tubulin positive twigs are found coursing through out the stroma. The boxed area is shown in higher magnification (insets) where β-III tubulin positive twigs are in close proximity to tumor cells. Arrows point out additional areas where β-III tubulin positive twigs are in close proximity to another tumor island. In the second case (**C**,**D**), tumor and stroma are clearly defined and β-III tubulin positive twigs are found throughout the stroma. The boxed area depicts higher magnification of these regions. In the third representative case (**E**,**F**), small tumor islands (T) are surrounded by stroma. The boxed regions are shown in higher magnification and illustrate the complexity of these sprouted twigs. Scale bar, 300 µm. n = 75 cases were analyzed.

Our new understanding of neuronal contributions to cancer progression, together with the poor prognosis associated with HGSOC, prompted us to define innervation in this tumor type. A total of 75 cases of HGSOC were studied by staining serial sections histologically with H&E and immunohistochemically (IHC) for β-III tubulin, a neuronal marker [39,40]. We paid particular attention to the presence and localization of β-III tubulin positive twigs in relation to tumor and stroma; representative examples are shown in Figure 1. In the first case, islands of tumor cells are surrounded by stroma (Figure 1A) with β-III tubulin positive twigs in close proximity to a tumor island (Figure 1B and high magnification inset). In another case, a large tumor is clearly demarcated from the stroma (Figure 1C) and β-III tubulin positive twigs are found coursing throughout the stromal compartment (Figure 1D, and high magnification inset). In the third example, tumor islands are again easily visible and surrounded by stroma (Figure 1E). Here, tumor cells are in close proximity to β-III tubulin positive twigs (Figure 1F and high magnification inset). Interestingly, we noticed that in many instances, β-III tubulin positive twigs localize near blood vessels (Supplementary Figure S1). We also noted that many HGSOC tumor cells themselves are positive for β-III tubulin; some samples exhibiting robust immunostaining (Supplementary Figure S2A, Figure 1D), others with variable staining (Supplementary Figure S2B) and still others predominantly negative for β-III tubulin (Supplementary Figure S2C, Figure 1F). It is important to note that twig and tumoral β-III tubulin staining are different. Twigs that are immune-positive for β-III tubulin are found coursing between cellular components of the stroma and tumor islands while tumor cell β-III tubulin staining is clearly cytoplasmic. This distinction enables us to focus our study on twigs. While the significance of β-III tubulin expression in tumor cells remains unclear, correlations with aggressive disease and poor survival exist [41]. Given our interest in tumor innervation, however, we focused only on β-III tubulin positive nerve twigs. 

Given the presence of twigs within HGSOCs, we wondered what types of nerves they were. Molecular studies demonstrate that HGSOCs are derived from fallopian tube secretory cells [9,12,42,43,44,45,46,47,48,49,50]; thus, normal tissue controls include normal fallopian tubes and ovaries. IHC staining shows that normal fallopian tube contains TH (sympathetic) positive nerve bundles (Supplementary Figure S3A, open arrowheads) that are negative for TRPV1 (sensory) and VIP (parasympathetic); scant single nerve fibers (Supplementary Figure S3A, β-III tubulin positive, small, filled arrowheads) are also evident. Normal ovary is similarly innervated with TH positive, TRPV1 and VIP negative nerve bundles (Supplementary Figure S3A, open arrowheads). While not all HGSOC cases harbor the same extent of twigs, the staining contrasts that of normal tissues; these twigs are TRPV1 positive but negative for TH and VIP (Supplementary Figure S3A, arrows). In addition, we immunofluorescently stained HGSOC cases for TRPV1 and β-III tubulin; their co-localization further indicates that HGSOCs are innervated by TRPV1 sensory neurons (Figure S3B). Positive controls for VIP, TRPV1 and TH IHC can be found in Supplementary Figure S4A–C. To further validate the presence of tumor-infiltrating twigs in HGSOC, we immunofluorescently stained patient samples for neurofilament, another neuronal marker (Supplementary Figure S4D). Since the type of innervation (sensory) in HGSOC differs from that in normal fallopian tube and ovary (sympathetic), these data suggest that HGSOCs obtain sensory nerves as a consequence of disease rather than by default. 

The presence of twigs within HGSOC is very similar to our published findings in head and neck squamous cell carcinoma (HNSCC) and cervical cancer patient samples. We wondered whether twigs are similarly present within the TME of other solid tumors. To test this, we surveyed a collection of cancers in a similar fashion. Ten samples per tumor type were scored for innervation by five independent scorers. Like HNSCC, cervical and HGOSC cancers, breast, prostate, pancreatic, lung, liver, and colon cancers harbor β-III tubulin positive nerve twigs (Supplementary Figure S5A–F). Importantly, these tumor-infiltrating twigs are seen as single fibers rather than established nerve bundles suggesting they are actively recruited to the tumor bed rather than pre-existing in the tissue. While β-III tubulin scoring of tumor-infiltrating twigs was variable, all tumor types analyzed were innervated (Supplementary Figure S5G). 

### 3.2. Syngeneic Model of HGSOC

**Figure 2:**
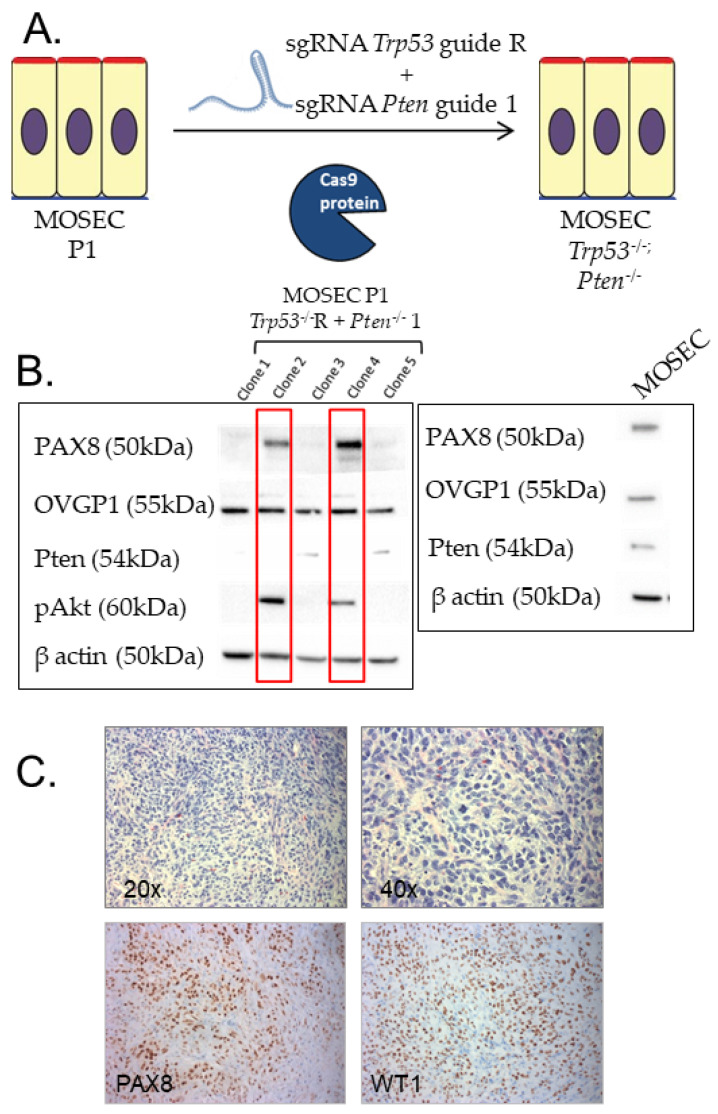
(**A**) Graphic illustration depicting methodology used to generate an MOSEC line deleted for *Trp53* and *Pten*. CRISPR-Cas9 was used to delete *Trp53* and *Pten* in early passage primary MOSECs. (**B**) Western blot analysis of CRISPR-Cas9 mediated knockout of *Trp53* and *Pten*. Five clones were evaluated for protein expression, including Pax8, Ovgp1, Pten, phospho-AKT, and β-actin. Loss of Pten protein was associated with acquisition of phospho-AKT in clones 2 and 4. MOSEC represents the parental murine oviductal secretory epithelial cells that was used for genome editing to generate the *Trp53; Pten* double knockout cell lines. (**C**) Morphology and immunophenotype of *Trp53; Pten* double knockout tumors. Top panel: H&E of tumors (20× and 40×) show a morphology consistent with a high-grade carcinoma. Lower panel: immunohistochemistry for Pax8 and WT1 (20×) shows that tumors retain lineage markers associated with high-grade serous carcinomas.

**Figure 3:**
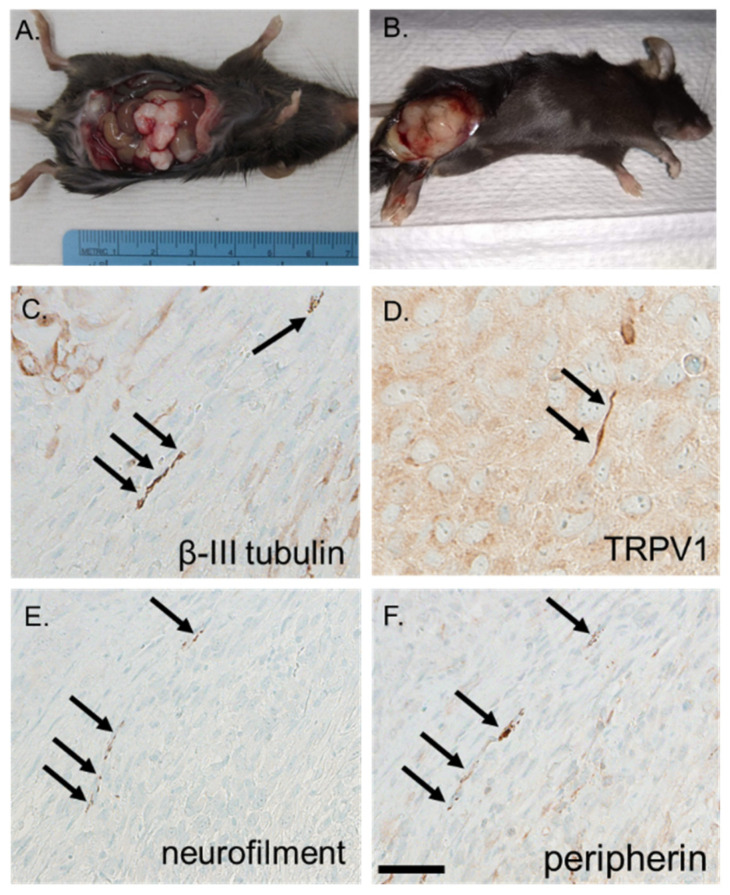
*Trp53*^-/-^ *Pten*^-/-^ syngeneic ovarian tumors grow in the peritoneal cavity (**A**) as well as subcutaneously (**B**). Immunohistochemical staining of subcutaneous *Trp53* ^-/-^ *Pten* ^-/-^ tumors demonstrates they harbor β-III tubulin (**C**), TRPV1 (**D**), neurofilament (**E**), peripherin (**F**) positive nerve twigs (arrows). Scale bar, 50 µm.

To assess and trace tumor-infiltrating twigs in HGSOC, we developed a syngeneic mouse model of the disease. In this model, murine oviductal secretory epithelial cells (MOSEC) from C57Bl/6 females harbor CRISPR-Cas9 mediated deletion of *Trp53* and *Pten*, commonly altered in HGSOC [51,52,53] (Figure 2A). Western blot analysis of positive clones validated their retained expression of lineage markers (Pax8, Ovgp1). As expected, loss of Pten resulted in phosphorylation and activation of Akt (Figure 2B). These cells generate tumors in mice that are Pax8 and WT1 lineage marker positive (Figure 2C) and grow following intraperitoneal (Figure 3A) as well as subcutaneous injection (Figure 3B). Notably, these murine tumors harbor β-III tubulin/TRPV1 positive nerve twigs (Figure 3C,D), identifying them as sensory in nature. Moreover, additional immunohistochemical (IHC) staining for the neuronal markers neurofilament and peripherin (Figure 3E,F) further validate the presence of nerves in these murine tumors. Taken together, these data support this as a faithful model of HGSOC and further support the presence of intra-tumoral nerves in this disease. 

### 3.3. Nerve Tracing in HGSOC

**Figure 4:**
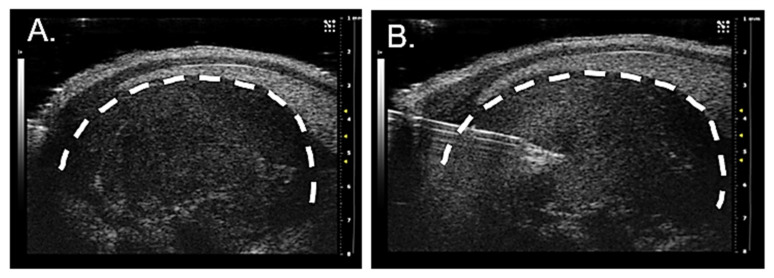
Representative ultrasound images of (**A**) transverse view of intraperitoneal tumor in lateral lower right quadrant of the abdomen showing skin line and fat under the skin. (**B**) Placement of the needle within the tumor for injection. Dotted line highlights tumor.

Retrograde axonal tracing was employed for visualizing tumor-infiltrating neurons. Given our findings demonstrating the presence of intra-tumoral twigs in many different cancers (Supplementary Figure S5) and that HGSOC innervation is sensory, differing from the sympathetic innervation endogenous to the tumor site (Supplementary Figure S3B), we wondered where the intra-tumor sensory twigs originate. For nerve tracing studies, female C57Bl/6 mice (n = 4) were implanted intraperitoneally with our murine model of HGSOC and tumor growth was monitored weekly by ultrasound. Four weeks post tumor implantation, anesthetized mice underwent ultrasound guided intra-tumoral injection with the fluorescently conjugated axonal tracer, WGA-A568 (Figure 4A,B). 

**Figure 5:**
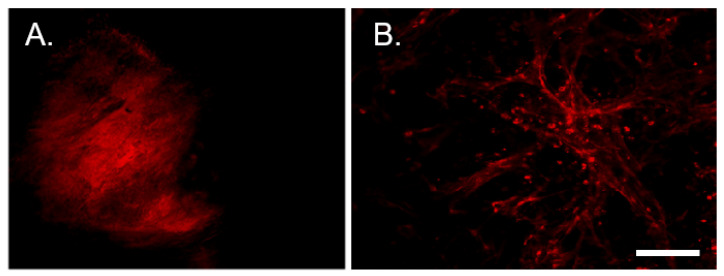
(**A**) Representative 4× confocal image of *Trp53* ^-/-^ *Pten* ^-/-^ tumor at the site of WGA-A568 (red) injection. (**B**) Tracer positive (red) intra-tumoral neuronal twigs distal to the injection site. Scale bar, 500 µm.

Tumors were permitted to continue growing for an additional 5-days post-tracer injection, after which animals were euthanized and tissues collected. Tumors and all associated tissues, including DRG were collected and analyzed for tracer fluorescence. Whole mount images of tumor taken near the site of tracer injection show bright tracer fluorescence (Figure 5A). Sections taken distal to the tracer injection site show tracer positive twigs (Figure 5B). The presence of tracer positive twigs indicates that their nerve terminals took up the tracer and retrogradely transported it such that their entire length is tracer positive. 

**Figure 6:**
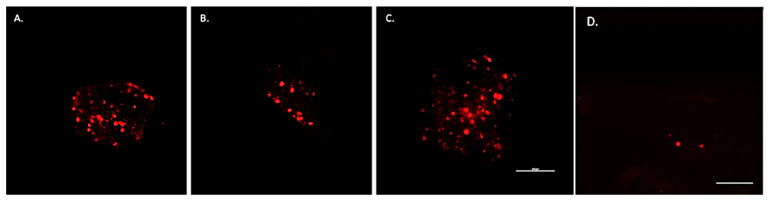
Representative 10× confocal images of whole mount DRG containing labeled neurons innervating HGSOC. (**A**) Labeled thoracic DRG neurons following WGA injections into abdominal HGSOC. (**B**) Labeled neurons of the jugular–nodose ganglia containing sensory peripheral neurons of the vagus nerve. (**C**) Labeled neurons of lumbar DRG (L4 spinal segment) following tracer injection into subcutaneous tumor in hind limb. (**D**) Labeling in a thoracic DRG following ip. injection of WGA into non-tumor bearing animal. Scale bar, 250 μm.

**Figure 7:**
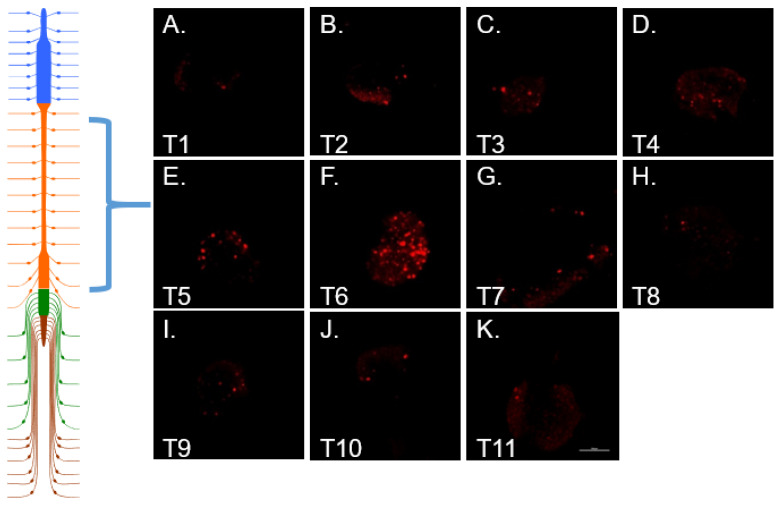
Cartoon of spinal cord and associated DRG. Panels (**A**–**K**) contain fluorescent images of WGA positive DRG from each of the thoracic segments (Scale bar = 250 μm, Cartoon was created using Motifolio Illustration Neuroscience Toolkit, https://www.motifolio.com. accessed on 1 September 2021).

In addition, retrograde transport of WGA also resulted in tracer fluorescence of ipsilateral neuronal somata within thoracic DRG and jugular–nodose ganglia identifying them as a source of intra-tumoral twigs (Figure 6A,B). There was some scattered labeling throughout the thoracic DRG with a precipitous increase concentrated on one spinal segment depending upon the location of the tumor in the peritoneal cavity (Figure 7). DRG from cervical, lumbar, and sacral spinal segments were unlabeled. In control animals in which tumors grew subcutaneously in the hindlimb, WGA fluorescence was exclusively present in lumbar DRG, L3-4 in particular (Figure 6C). Labeled cells were dispersed throughout each labeled ganglion. 

As an additional control, non-tumor bearing animals were injected intra-peritoneally with the same amount of WGA and tissues similarly harvested five days later. Since the peritoneal cavity is normally innervated by sensory and autonomic fibers, tracer labeling was expected. Interestingly, in these controls WGA labeling was widespread and bilateral. This was in stark contrast to the ipsilateral labeling that occurs following intra-tumoral tracer injections. Moreover, tracer positive neurons from tumor-bearing animals were restricted to specific segments of the thoracic ganglia with one segment harboring the strongest labeling. In addition, within tracer positive ganglia from tumor-bearing animals, somata were intensely bright suggesting a high density of their terminals present at the tumor bed (the site of tracer injection). In contrast, in control non-tumor bearing animals, tracer labeling was not focused to specific ganglia and positive somata harbored a weak tracer signal suggesting widespread uptake and, thus, dilution of WGA from the peritoneum (Figure 6D). Taken together, these data indicate that abdominal HGSOCs are specifically innervated by loco-regional sensory nerves that originate from peripheral ganglia. Moreover, the stark contrast in the location of tracer (bilateral vs. ipsilateral) and the robustness of its signal (diffuse vs. strong) between non-tumor bearing and tumor-bearing animals indicates that ganglionic labeling following intra-tumoral WGA injections does not occur due to diffusion of the tracer into the peritoneal cavity. Instead, the labeling occurs from the retrograde uptake of WGA from nerve terminals concentrated within the tumor bed. 

**Figure 8:**
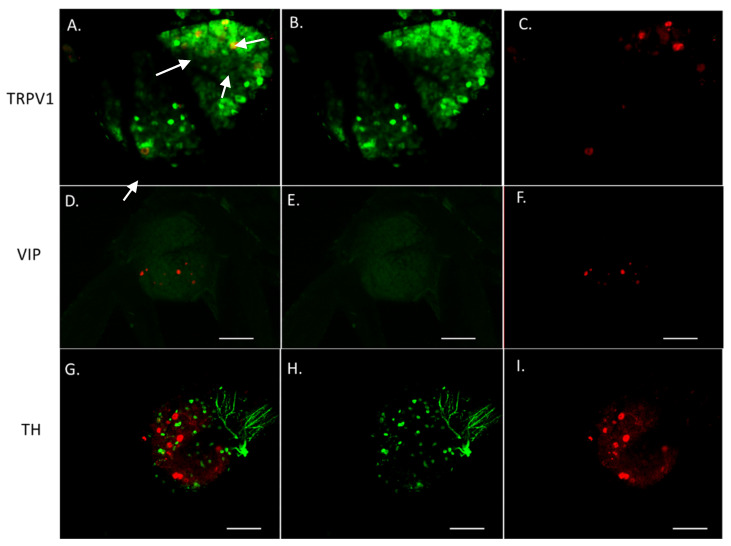
Representative confocal images of whole mount DRG containing WGA labeled (red) neurons innervating HGSOC tumor with TRPV1 (green, (**A**–**C**)), VIP (green, (**D**–**F**)) or TH (green, (**G**–**I**)) staining. Expression of TRPV1 was observed in tracer labeled thoracic DRG neurons (**A**, arrows) whereas no TH or VIP staining was observed in tracer labeled neurons (**D**,**G**). Scale bar = 100 μm.

To define what type of neurons (sensory, sympathetic, parasympathetic) innervate murine HGSOCs, tracer positive DRG were immunofluorescently stained as follows. TRPV1 was used as a sensory marker, tyrosine hydroxylase (TH) was used as a sympathetic marker and vasoactive intestinal polypeptide (VIP) was used as a parasympathetic marker. Approximately 30% of tracer positive DRG neurons from tumor-bearing animals co-labeled with TRPV1 (Figure 8A), the majority were TRPV1 negative (Figure 8B,C). Moreover, intra-tumoral neurons do not co-label with TH or VIP suggesting they are not sympathetic or parasympathetic in nature (Figure 8D–I). Future studies will focus on utilizing additional markers to further define innervation in HGSOC. 

## 4. Discussion

Our data support the literature demonstrating that innervation is a common feature for multiple cancers [13,17,18,54,55,56,57,58,59]. Recent findings of neural activity recorded in vivo from murine breast cancer further attest not only to the presence of neurons in tumors but to their function within the tumor bed [54]. Here, we show that, similar to head and neck and cervical cancers, breast, prostate, pancreatic, lung, liver, ovarian and colon cancers contain β-III tubulin positive nerve twigs. The IHC staining of patient tumors show that, in contrast to the sympathetic innervation of the fallopian tubes and ovaries, HGSOC tumors are innervated by sensory (TRPV1 positive) nerves. 

To further assess innervation of HGSOC, we developed a syngeneic mouse model of the disease and investigate the source of these tumor-infiltrating nerves. Our tracing of intra-tumoral nerve terminals to peripheral ganglia clarifies the innervation of HGSOC via recruitment and sprouting from existing local peripheral nerves. Several neurites sprout from each peripheral process resulting in extensive tumor innervation. These sprouted twigs retrogradely transport the WGA tracer and label a relatively smaller number of DRG and vagus somata. The induction of twig sprouting from existing fibers is similar to axonal sprouting associated with nerve injury [60]. We hypothesize that the TME mimics an injury environment. These signals, together with those released from the tumor cells, likely signal loco-regional axons to sprout and innervate the tumor. While this hypothesis remains to be fully tested, our data support such a mechanism. This finding further indicates that the sensory nerves detected within the tumor tissue arise from extrinsic innervation from dorsal root ganglia and the vagus nerve. The site of implantation and tumor formation dictates the recruitment of regional nerves along spinal segments. For example, injection of tracer into hind limb tumors preferentially labels lumbar segments, whereas intraperitoneal injection into abdominal tumors labels thoracic segments, with different thoracic levels demonstrating tracer uptake depending upon the tumor location within the abdomen. Tracer-labeled perikarya were present throughout the ipsilateral DRG and nodose ganglia with no topographical localization of labeled cells within any ganglia. Interestingly, immunofluorescent staining for TRPV1 shows that approximately 30% of tracer positive twigs also express TRPV1. This is in contrast to the IHC data from patient samples which supports that the majority of the intra-tumoral twigs are TRPV1 expressing. While this disparity may reflect differences in antibody binding between FFPE and perfusion-fixed tissue or differences between mouse and human tumors, an alternative possibility is more likely. A recent study analyzing mouse and human DRG shows that approximately 32% of neurons in murine DRG are TRPV1 positive while in humans, TRPV1 expressing somas make up 74% of the DRG [58]. Regardless of the reason for the difference we see in TRPV1 positivity of intra-tumoral nerves, our analysis of patient and mouse tumor samples indicates that HGSOCs are innervated and that at least one third of this innervation is sensory in nature.

The majority of the nerve fibers in the vagus nerve are afferent sensory nerves communicating the state of the periphery to the brain. Vagal stimulation has recently been shown to drive intra-tumoral electrical activity [54]. In addition, vagotomy has been shown to decrease tumor growth in a mouse model of intestinal cancer suggesting this input could act to promote carcinogenesis [55]. However, pharmacological inactivation of the vagus or vagotomy enhances metastasis in an orthotopic model of breast cancer [56,57]. This may be due to differences in surgery (mid-neck versus sub-diaphragmatic vagotomy), cancer cell types, and/or tumor location. Together, these data demonstrate that vagal afferents provide input to the TME, although the role of this active vagal sensory input to the TME remains unclear. Future studies will define whether modulation of vagal input to HGSOC influences disease progression. 

Following intra-tumoral injection of axonal tracer, we found labeling within regional DRG neurons (which reside along the spinal cord) which contain the somata of primary sensory neurons that are critical structures in sensory transduction. Pain is a common problem among those with advanced ovarian cancer, suggesting that sensory innervation may occur at later stages of growth and contribute to this pain signaling. In the present study, tracer injection occurred four weeks following implantation, when tumors are relatively large. However, innervation may occur at earlier stages and may change during disease progression or occur at a specific growth point. Future studies will examine earlier time points in tumor growth to characterize the timeline of innervation in HGSOC. It is important to note that labeled DRG neurons may not be the direct source of innervation as it remains possible that intermediary neurons within the tumor contact these DRG fibers.

We noticed that twigs were generally found near blood vessels. This is not entirely surprising as, during development, axons and blood vessels travel together. We hypothesize that signals released from the tumor diffuse loco-regionally. Once these signals are received by nearby axons, they respond by sprouting twigs that extend into the tumor bed. Since axons track near vessels, tumor-induced twig sprouting occurs at these sites. While some patients are treated with VEGF inhibitors to block tumor angiogenesis, based on our current findings, it is unclear whether such treated tumors would demonstrate less innervation. Additional studies would need to address this possibility.

In the present study we did not assess whether there are alterations in DRG neuron activity as a consequence of their terminals invading the TME. Changes to the activity of even just a few DRG could broadly impact sensory-sympathetic activity. In preclinical pain models, sympathetic sprouting in DRG has been observed and sympathetic stimulation modulates DRG neuronal activity [59]. Moreover, DRG stimulation can also influence the activity of sympathetic nerves [61]. In addition, the majority of DRG neurons undergo depolarization when the axon of a neighboring DRG neuron within the same ganglion is stimulated [62]. Taken together, these studies suggest that altered signaling within DRG innervating the TME could have broad impact on regional neuronal activity through signal amplification or sympathetic and sensory interactions. Whether this impacts tumor growth or, alternatively, whether the tumor itself modulates this neuronal activity, remains to be defined. Future studies will investigate this neuronal plasticity associated with tumor innervation and identify molecular adaptations within tumor-projecting sensory neurons. As we gain an increased molecular understanding of intra-tumoral nerves and the neural circuits they impact, we will be better equipped to identify and therapeutically target these novel components of the TME.

## Data Availability

No new datasets were generated in this study.

## Supplementary Materials

The following are available online at https://www.mdpi.com/article/10.3390/cells10123491/s1, Figure S1: Twigs and blood vessels; Figure S2: Tumor expression of β-III tubulin; Figure S3: HGSOC innervation. Figure S4: Positive controls for IHC and additional neuronal marker; Figure S5: Innervation in solid tumors.
