# Supplementary material for: Intra-Tumoral Nerve-Tracing in a Novel Syngeneic Model of High-Grade Serous Ovarian Carcinoma
Source: Cells. 2021 Dec 10;10(12):3491. doi: 10.3390/cells10123491 (PMC8699855; doi:10.3390/cells10123491)
Supplement: Supplementary file 1 [file cells-10-03491-s001.zip › cells-1384143-supplementary.pdf]

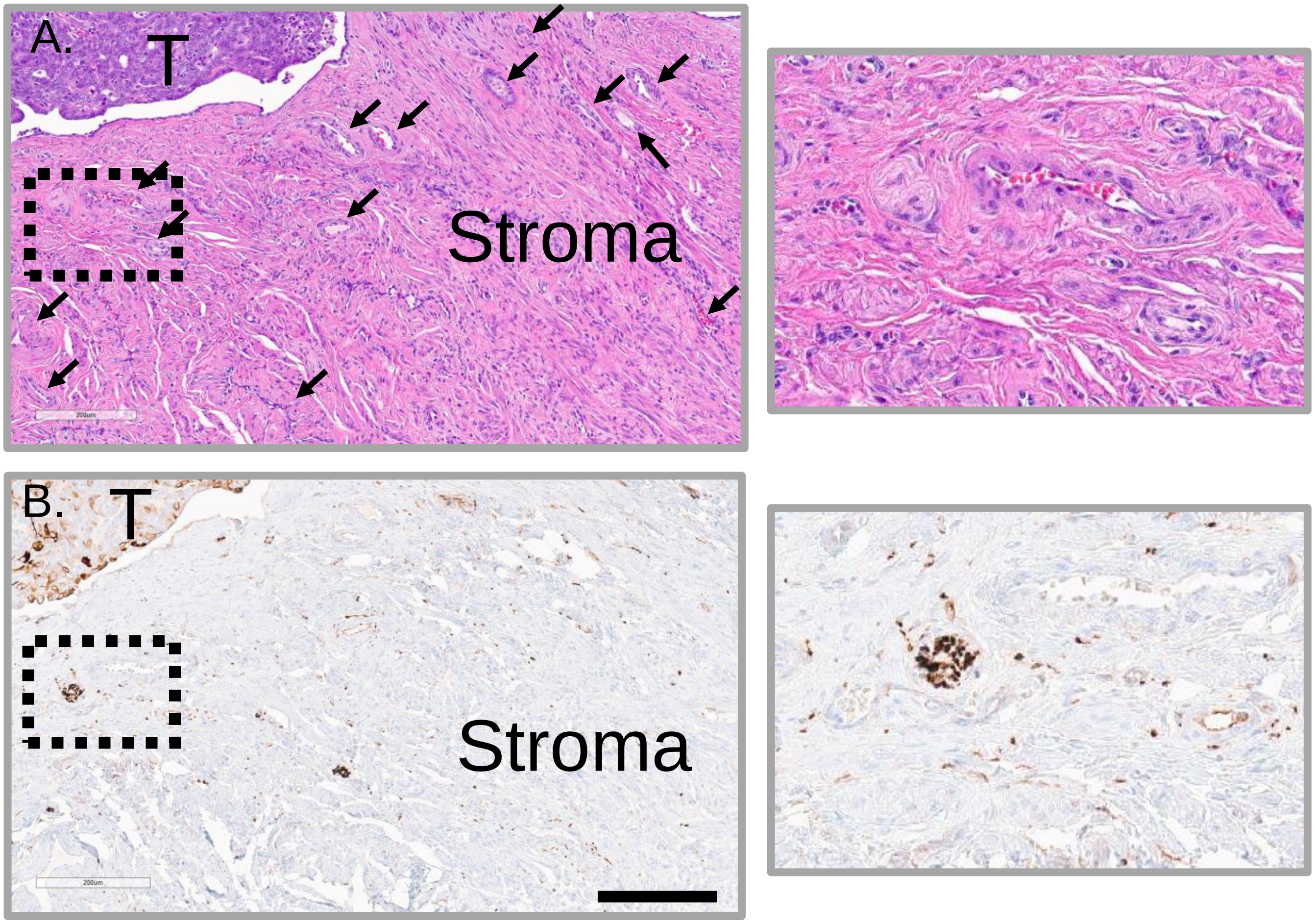

Supplemental Figure S1. Twigs and blood vessels.
Representative example of HGSOC. Serial sections stained with H&E (A) or immunohistochemically stained for β-III tubulin (brown) (B). Tumor (T) is labeled and surrounded by stroma. Throughout the stroma are many blood vessels (arrows). Boxed areas of interest are shown in higher magnification. Scale bar, 500 µm.

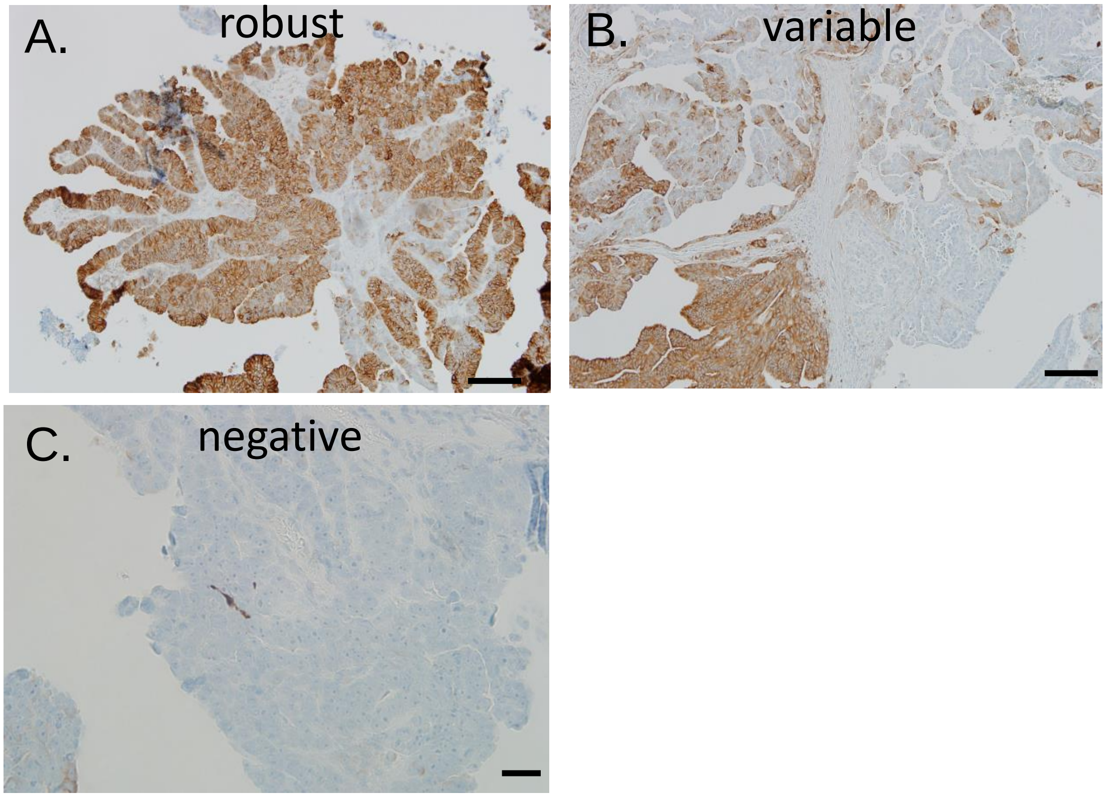

Supplemental Figure S2. Tumor expression of β-III tubulin. Representative examples of HGSOC cases immunohistochemically stained for β-III tubulin (brown) in which tumor cells exhibit robust (A), intermediate (B) or no (C) staining. Scale bar, 10 μm.

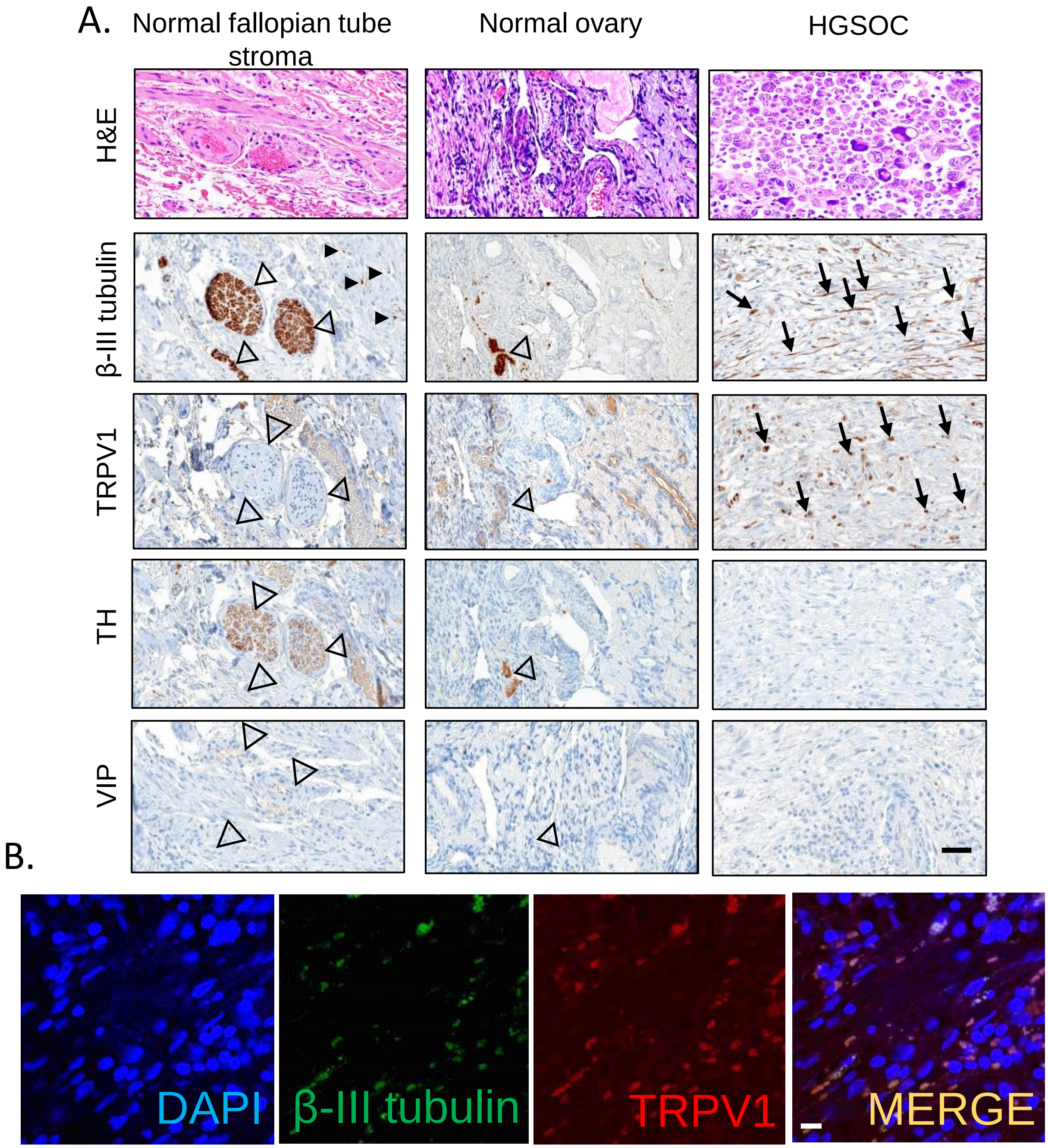

Supplementary Figure S3. HGSOC innervation. (A) Representative bright field images of normal fallopian tube (n=10), normal ovary (n=10) and HGSOC samples (n=75) histochemically stained with hematoxylin and eosin or immunohistochemically stained as indicated. Large arrowheads, nerve bundles; small black arrowheads and small arrows, nerve twigs; scale bar, 10μm. (B) Representative *en face* confocal images of HGSOC sample double immunofluorescently stained as indicated. N=8 patient samples immunofluorescently stained. Scale bar, 10μm. Brightness was modified uniformally across all images.

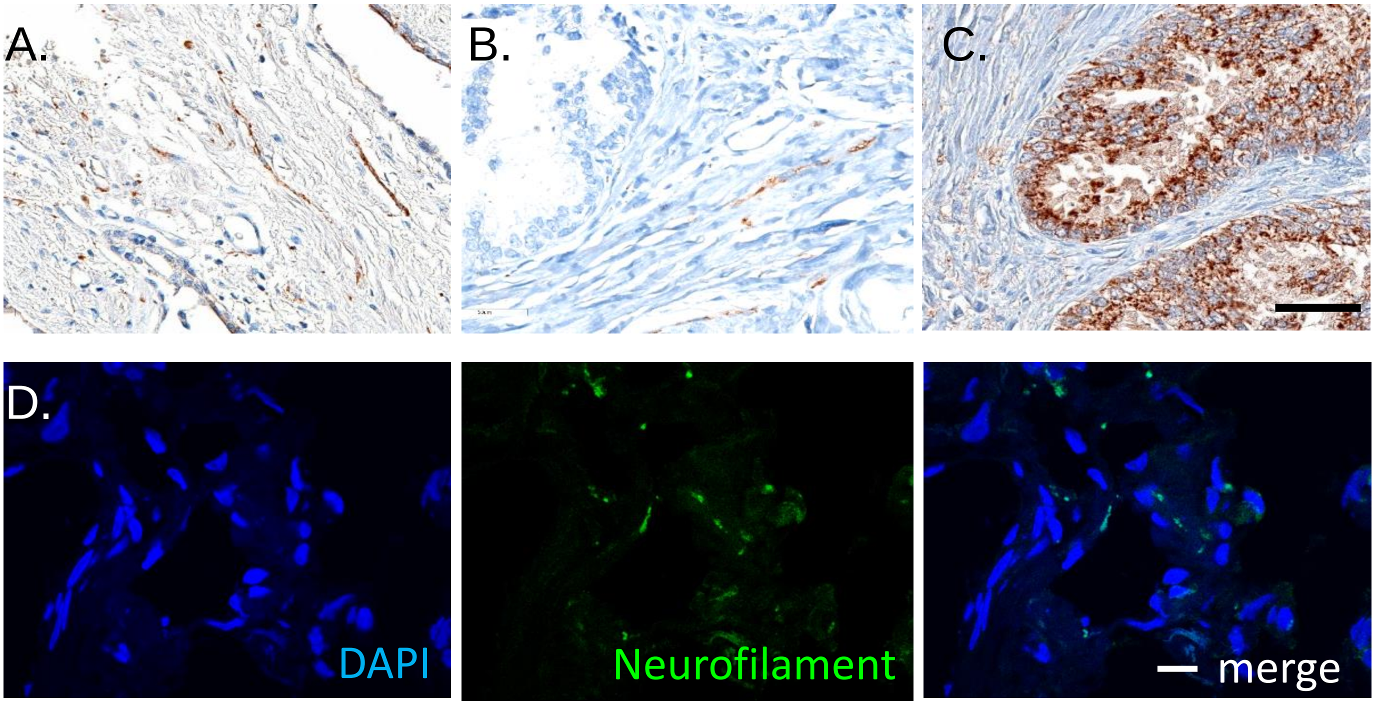

Supplemental Figure S4. Positive controls for IHC and additional neuronal marker. Positive tissues for IHC staining as follows. Human prostate cancer as positive control for (A) VIP, (B) tyrosine hydroxylase, and (C) TRPV1. Scale bar, 50 µm. D) Human HGSOC immunofluorescently stained for neurofilament (green); counterstained with DAPI (blue). Scale bar, 10µm.

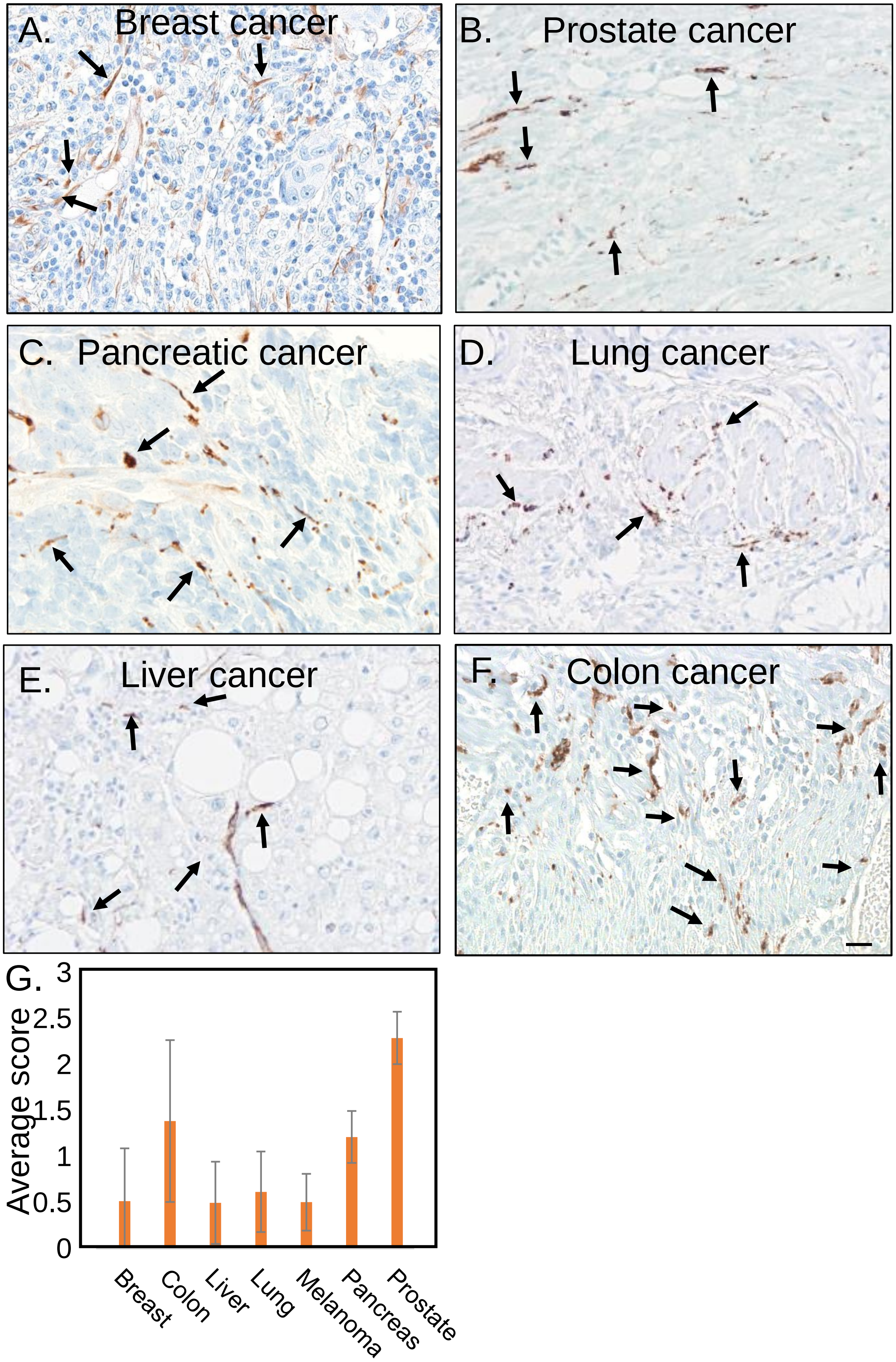
A. Breast cancer
B. Prostate cancer
C. Pancreatic cancer
D. Lung cancer
E. Liver cancer
F. Colon cancer
G.
Average score
0
0.5
1
1.5
2
2.5
3
Breast
Colon
Liver
Lung
Melanoma
Pancreas
Prostate

Supplementary Figure S5. Innervation in solid tumors. (A-G) Bright field images of indicated tumors IHC stained for β-III tubulin (brown, arrows; n=10 tumors/type except ovarian with n=75). Light blue, counterstain. Scale bar, 20μ m. (H) Average innervation score (β-III tubulin)/tumor type. All patient samples were scored for nerve twigs by five independent evaluators, each scored 5 random 20X magnification images/ sample. Scoring averages are graphed; standard deviation as error bars.
